# Organization of Nucleotides in Different Environments and the Formation of Pre-Polymers

**DOI:** 10.1038/srep31285

**Published:** 2016-08-22

**Authors:** Sebastian Himbert, Mindy Chapman, David W. Deamer, Maikel C. Rheinstädter

**Affiliations:** 1 Department of Physics and Astronomy, McMaster University, Hamilton, L8S 4M1, Canada; 2 Origins Institute, McMaster University, Hamilton, L8S 4M1, Canada; 3 Department of Experimental Physics, Saarland University, 66123 Saarbrücken, Germany; 4 Department of Biomolecular Engineering, University of California, Santa Cruz, 95064, USA

## Abstract

RNA is a linear polymer of nucleotides linked by a ribose-phosphate backbone. Polymerization of nucleotides occurs in a condensation reaction in which phosphodiester bonds are formed. However, in the absence of enzymes and metabolism there has been no obvious way for RNA-like molecules to be produced and then encapsulated in cellular compartments. We investigated 5′-adenosine monophosphate (AMP) and 5′-uridine monophosphate (UMP) molecules confined in multi-lamellar phospholipid bilayers, nanoscopic films, ammonium chloride salt crystals and Montmorillonite clay, previously proposed to promote polymerization. X-ray diffraction was used to determine whether such conditions imposed a degree of order on the nucleotides. Two nucleotide signals were observed in all matrices, one corresponding to a nearest neighbour distance of 4.6 Å attributed to nucleotides that form a disordered, glassy structure. A second, smaller distance of 3.4 Å agrees well with the distance between stacked base pairs in the RNA backbone, and was assigned to the formation of pre-polymers, *i.e.*, the organization of nucleotides into stacks of about 10 monomers. Such ordering can provide conditions that promote the nonenzymatic polymerization of RNA strands under prebiotic conditions. Experiments were modeled by Monte-Carlo simulations, which provide details of the molecular structure of these pre-polymers.

There is no satisfying explanation for how the earliest forms of cellular life first emerged on Earth at least 3.5 billion years ago^1^, and this compelling question has driven laboratory studies since the discovery of the genetic code. The basic molecular components of life can be categorized in three different groups: boundary forming amphiphilic molecules of cell membranes needed to confine cellular components, enzyme catalysts to guide and facilitate essential metabolic reactions, and information-storing molecules, such as RNA or DNA^2^,^3^,^4^.

A testable hypothesis is that an early RNA-based life form may have preceded the appearance of the DNA/protein code that is universal to life on Earth^5^,^6^. If so, the nonenzymatic polymerization and replication of RNA would be a critical step in the emergence of simple cellular life from prebiotic chemistry^7^. The polymerization of nucleotides is a condensation reaction in which phosphodiester bonds are formed. This reaction cannot occur in aqueous solutions, but guided polymerization in an anhydrous or confined environment could promote a non-enzymatic condensation reaction in which oligomers of single stranded nucleic acids are synthesized^8^,^9^.

The idea of guided polymerization was first proposed forty years ago^6^,^10^,^11^, and it was recently shown that RNA-like polymers can be synthesized non-enzymatically in conditions simulating a prebiotic hydrothermal site undergoing cyclic fluctuations in hydration^12^. The cycles involved dehydration at elevated temperature in the presence of promoting agents. For example, a lipid matrix served to concentrate and organize the mononucleotides and bring the functional groups, the sugar and the ribose, in close proximity^13^. A second promoter was reported by Da Silva *et al.*^14^ who achieved even higher yields of polymers when the mononucleotides were mixed with inorganic salts, such as NaCl, KCl and NH_4_Cl.

To address the effect of confinement and anhydrous conditions in the polymerization reaction, we investigated the organization of adenosine (AMP) and uridine monophosphate (UMP) in different environments. Toppozini *et al.*^13^ observed that lipid matrices organized nucleotides, such as 5′-adenosine monophosphates (AMP), into nanometer sized 2-dimensional crystallites. We chose to use a 1:1 mole ratio mixture of AMP and UMP in order to allow the possibility that hydrogen bonded base pairing could further enhance self-assembly of stacked bases in different conditions.

To elucidate the effect of a nanoscale confinement, AMP/UMP complexes were studied in a lipid matrix made of dimyristoylphosphatidylcholine (DMPC, Fig. 1a.) As a control, films of AMP/UMP nucleotides were prepared as thin, nanometer thick films on silicon wafers in the absence of organic molecules or anhydrous or charged surfaces (Fig. 1b). Inorganic salts also provide confinement under anhydrous conditions because solutes like nucleotides are excluded from salt crystals during drying and become concentrated as thin films on the crystal surfaces (Fig. 1c). Finally, mineral surfaces, such as Montmorillonite clay with their nanoporous structure also serve to confine chemically activated nucleotides^15^,^16^ (Fig. 1d). The charged clay surface attracts the nucleotides and the increased local concentration may promote ester bond synthesis between nucleotides during dehydration.

Two-dimensional X-ray diffraction was used to study the molecular organization of the nucleotides, as shown schematically in Fig. 1e. We find two distinct nucleotide distances in all systems and assigned a distance of 4.6 Å to nucleotides that form a disordered, glassy structure, and a smaller distance of 3.4 Å corresponding to stacks of nucleotides at the distance between stacked base pairs. These pre-polymers are likely to be in the form of extensive linear stacks in which ribose and phosphate groups come into close contact. As a result, the monomers can be linked into long strands by ester bond synthesis when the activity of water is sufficiently low^14^.

## Materials and Methods

### Preparation of the AMP/UMP complexes

The free acid form of 5′-adenosine monophosphate (5′-adenylic acid, C_10_H_14_N_5_O_7_P) powder and 5′-uridine monophosphate (5′-uridylic acid, C_9_H_13_N_2_O_9_P) powder were added to ultra pure water in 10 mM concentrations and heated in a water bath until completely dissolved. The AMP/UMP solutions and DMPC, NH_4_Cl were then mixed in molar ratios of 1:1.

All complexes were prepared on single-side polished silicon wafers. 100 mm diameter, 300 *μ*m thick silicon (100) wafers were pre-cut into 10 × 10 mm^2^ chips. The wafers were first pre-treated by sonication in dichloromethane (DCM) at 40 °C for 25 minutes to remove all organic contamination and leave the substrates in a hydrophobic state. Each wafer was thoroughly rinsed three times by alternating with ~50 mL of ultra pure water with a resistivity of 18.2 MΩ cm and methanol.

A silicon chip was placed on a hot-plate and heated to 85 °C. A 50 *μ*L aliquot of the final suspension was pipetted onto the wafer, forming a ~5 mm drop that completely dried in ~1 minute. Samples of the pure matrices were prepared from 10 mM dispersions of the respective materials for comparison.

To fabricate thin nucleotide films, nucleotide solutions were applied as thin films onto silicon wafers. The thickness of the films was determined through optical analysis to ~200 nm.

Multilamellar solid-supported lipid bilayers were prepared by vesicle fusion^17^,^18^,^19^. Small lipid vesicles (liposomes) were prepared by dispersing 1,2-dimyristoyl-sn-glycero-3-phoshatidylcholine (DMPC) in ultra pure water to produce concentrations of 10 mM. The milky solution, which initially contained multi-lamellar vesicles (MLVs), was sonicated for 15 minutes until the solution became transparent, indicating that small unilamellar vesicles (SUVs) formed. Care was taken to maintain the nucleotide solution at a temperature of at least 30 °C during the deposition process and storage to keep the bilayers in their fluid phase above the phase transition temperature (T_*m*_) of 23.9 °C^20^. We note that while DMPC is a standard phospholipid and often used in experiments and computer models, it is a modern diacylphospholipid and unlikely to have been present in large quantities in abiotic systems. In fact, complex amphiphiles were likely not present on the prebiotic Earth but represented by simpler amphiphilic molecules, such as fatty acids^21^.

**Figure 1:**
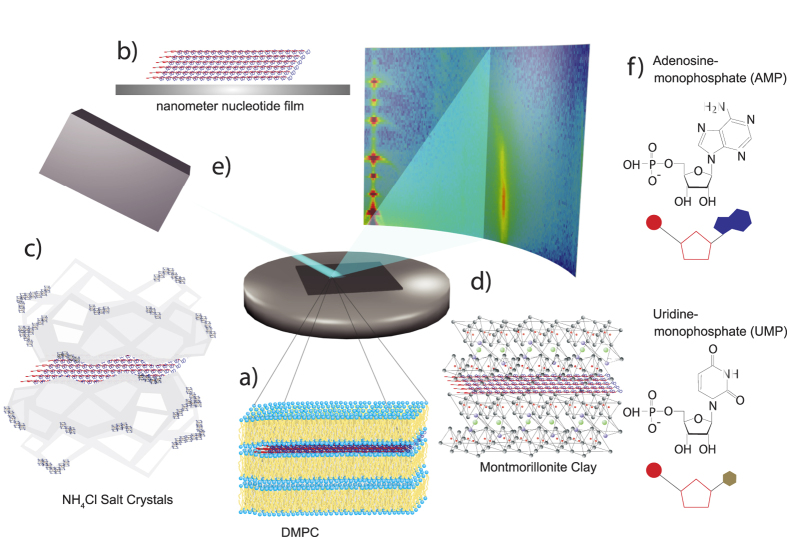
Schematics of systems studied, the scattering geometry, and AMP and UMP molecules. Four environments were investigated: (**a**) nucleotides confined between lipid bilayers made of DMPC, (**b**) thin films of nucleotides applied on silicon wafers, (**c**) nucleotides in NH_4_Cl, and (**d**) nucleotides confined in Montmorillonite clay. (**e**) Two-dimensional X-ray diffraction maps were recorded to capture signals of the pure materials and signatures of the organization of the nucleotides. (**f**) Structures of the adenosine monophosphate (AMP) and uridine monophosphate (UMP) molecules.

During drying, the vesicles first form a concentrated gel on the silicon surface, then undergo further drying and fusion into multilamellar structures parallel to the plane of the silicon surface. Previous studies^22^,^23^ demonstrated that small solutes, such as AMP, are confined between alternating bilayers because the empty interiors of the vesicles exclude solutes during the fusion process. We envision that a typical structure consists of a thin layer of AMP and UMP molecules separated from the next AMP/UMP layer by two lipid bilayers (Fig. 1a).

To study the effect of inorganic salts, NH_4_Cl with a purity of >99.5% was dissolved in ultra pure water at a concentration of 10 mM. The solution was vortexed and heated to 50 °C to ensure that all the salt has dissolved.

For the clay complexes, Montmorrilonite K 10 clay (Al_2_O_9_Si_3_, CAS Number: 1318-93-0) was obtained as powder from Sigma (Product Number 69866, pH 2.5–3.5, surface area 250 m^2^/g). The powder was added to ultra pure water at a concentration of 2.82 mg/ml. The solution was thoroughly vortexed and heated to ~70 °C resulting in a slightly milky solution. We note that different Montmorillonite clays may have different elemental compositions and contain different amounts of iron, magnesium, and aluminum. The findings in this work are, therefore, strictly valid only for the particular clay used. When applied to the silicon chips, a smooth and uniform clay film was formed.

### X-ray diffraction experiment

X-ray scattering data were obtained using the Biological Large Angle Diffraction Experiment (BLADE) in the Laboratory for Membrane and Protein Dynamics at McMaster University. BLADE uses a 9 kW (45 kV, 200 mA) CuK*α* rotating anode at a wavelength of 1.5418 Å. Both source and detector are mounted on movable arms such that the membranes stay horizontal during the measurements. Focusing multi-layer optics provides a high intensity parallel beam with monochromatic X-ray intensities up to 10^10^ counts/(mm^2^ s). This beam geometry provides optimal illumination of the solid supported samples to maximize the scattering signal. A sketch of the scattering geometry is shown in Fig. 1e. Note that there is no risk of sample damage using this in-house technique because of the large beam size and relatively low intensity of the X-ray beam, as compared to synchrotron sources.

The result of such an X-ray experiment is a 2-dimensional intensity map of a large area of the reciprocal space, as sketched in Fig. 1e. The corresponding real-space length scales are determined by *d* = 2*π*/|*Q*| and cover length scales from about 2.5 to 100 Å (Q is the total scattering vector). All scans were measured at 28 °C and 50% relative humidity (RH) hydration. As depicted in Fig. 1e, the wafers were oriented in the X-ray diffractometer, such that the *q*_‖_-axis probed lateral structure, parallel to the wafer surface, and the perpendicular axis, *q*_*z*_, probed out-of-plane structure, perpendicular to the substrate.

The atomic structure of the AMP molecules was taken from^24^; the structure of crystalline AMP from^25^. Crystal structure of UMP was taken from^5^. Molecular structures of AMP and UMP molecules and their cartoon representations are displayed in Fig. 1f.

We note that this type of experiment cannot be compared to protein crystallography, where atomic resolution protein structures are determined from protein crystals. The AMP and UMP molecules in our experiment are embedded in different complexes. The corresponding structures are inherently disordered, which leads to a strong suppression of higher order Bragg peaks in the experimental data inhibiting a direct Fourier transformation. We used X-ray diffraction in this experiment to detect the signatures of nucleotides in the different matrices.

Two-dimensional X-ray diffraction was collected for all systems and is shown in Fig. S1 in the Supplementary Material. Data for the pure matrix (except for the films), the matrix mixed with AMP, UMP and AMP/UMP are shown. As will be discussed below, a series of well defined Bragg intensities is observed for NH_4_Cl, Montmorrilonite clay and DMPC bilayers. The presence of nucleotide led to additional scattering features.

The experimental errors were determined as follows: Errors for peak positions, peak width and peak height are determined as the fit standard errors, corresponding to 95% confidence bounds, equivalent to 2 standard deviations, σ. Errors for calculated parameters, such as peak area, were then calculated by applying the proper error propagation.

### Computer modeling

Organization of nucleotides in the different environments was simulated by Monte-Carlo methods using the Gillespie algorithm. Organization of nucleotides in the nucleotide stacks was modeled by the following set of rate equations:


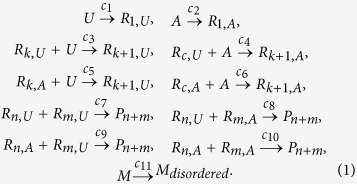


The first equations describe the addition of additional mononucleotides to the pre-polymers *R*_*μ*_. *c*_1_ and *c*_2_ are the rate constants for a nucleotide to become the nucleus of a pre-polymer. As the pre-polymers may be ended by *A* or *U*, there are different rate constants for adding additional *A* or *U* monomers, as described by the constants *c*_3_ to *c*_6_. *c*_7_ to *c*_10_ include the possibility of two *R*_*μ*_ combining into longer stacks *P*_*μ*_. While the first 10 equations describe the formation of the pre-polymers, the last equation describes the transition of a monomer *M* into a disordered state. The constants *c*_*μ*_ with *μ* = {1, 2, …, 11} characterize the speed of each formation. Note that these parameters are, therefore, not directly comparable to a classical reaction constant.

A two monomer system was implemented based on the rate equations *R*_*μ*_ in Eq. (1) to analyze potential final polymer configurations. The algorithm of the Gillespie method randomly selects in every Monte-Carlo step one of the possible reactions *R*_*μ*_, and the increment of the next time step *τ*^26^,^27^. The probability distribution of the random numbers can be written as:


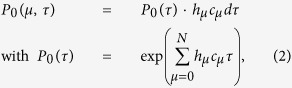


where *c*_*μ*_ are constant parameters as specified above, and *h*_*μ*_ is defined as the number of distinct molecular reactant combinations for reaction *R*_*μ*_ found to be present in Volume *V* at time *t*. The reaction constants *c*_3_, *c*_4_ and *c*_5_, and *c*_6_ were determined directly from the experiment, as will be described below. The remaining constants could not be determined from the experiment, however, were modified until the simulated length distribution was found to match the experimental results.

An initial system of 10000 monomers was used in the simulations. The algorithm aborts when no more free monomers are available and no more reactions can occur. Monte-Carlo simulations are statistical methods and the result of one single simulation is liable to statistical noise. Hence, each simulation was run 10000 times.

We note that these simulations do not describe an equilibrium state. Using the idea of wet and dry cycles, there would be a high diffusivity of the molecules in the wet state, while molecular motion is basically suppressed in the dry state. The number of reaction partners is then limited by the molecules in close proximity. If all molecules have reacted according to the rate equations, the simulation stops. New reaction partners are then brought in through a wet cycle and the structure formation starts over in the next dry cycle^21^. The above simulations thus describe formation of structures in a single dry cycle. We argue that the rate of formation of ordered stacks in this kinetic trap is drastically enhanced and can result in the accumulation of pre-polymers over time.

## Results

### Nucleotide structures

**Figure 2:**
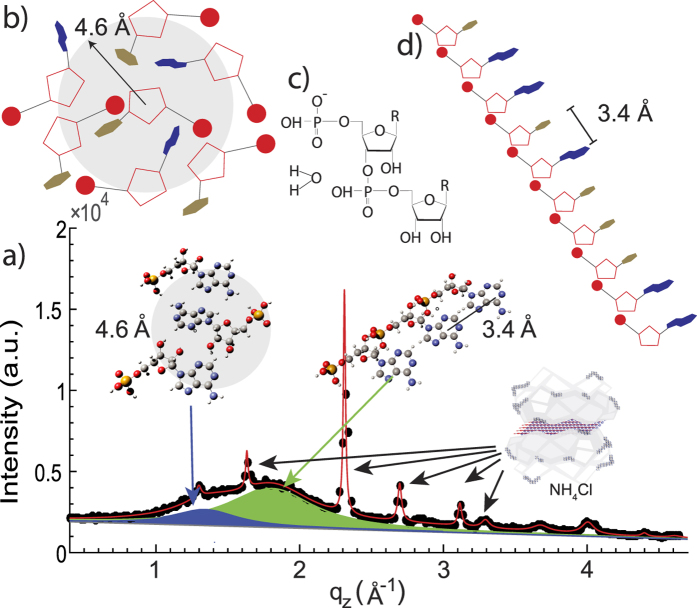
Organization of nucleotides and corresponding scattering patterns. (**a**) Two distances are observed in the diffraction experiments as scattering contributions at *Q* values of 1.34 Å^−1^ (corresponding to 4.6 ± 0.2 Å) and 1.85 Å^−1^ (corresponding to 3.4 ± 0.1 Å). The diffraction pattern observed in NH_4_Cl is shown as an example. (**b**) Nucleotides forming a disordered structure with an average distance between nucleotides of 4.6 Å. (**c**) Formation of a 3′ bond between nucleotides is a condensation reaction, where 1 water molecule is produced. (**d**) Nucleotides at a distance of 3.4 Å are indicative of nucleotides forming stacks at the base pair distance, so-called pre-polymers.

A typical diffraction pattern is shown in Fig. 2a, using NH_4_Cl mixed with AMP/UMP as an example. The pattern consists of a number of narrow signals from the salt matrix and 2 broader signals, which are observed in the presence of nucleotides, only. These signals were observed in all matrices in the presence of nucleotides and are well described by 2 Lorentzian distributions. By fitting width and position of the corresponding Lorentzian peaks, the two signals occur at distances of 4.6 ± 0.2 Å (1.34 Å^−1^) and 3.4 ± 0.1 Å (1.85 Å^−1^) .

Based on the observation by Toppozini *et al.*^13^ these distances can be modeled by 2-dimensional nucleotide arrangements, where the AMP and UMP molecules take a flat position between the bilayers. The nucleotides form a ~2.7 Å thick layer, which has been modeled with the ribose ring and the phosphate group aligning parallel to the bilayers. Such a 2-dimensional arrangement is plausible when the nucleotides are confined between lipid bilayers to minimize the free energy, which includes contributions from nucleotides and elastic energy of the bilayers. While in the 3-dimensional crystal structures of nucleotides, the ribose and the nucleobase are not coplanar, rotations of the nucleobase were reported in the case of AMP, such that the molecules in confinement may take a different, more 2-dimensional structure^25^. If the nucleotides formed complexes with the matrix, the structure and the corresponding diffraction pattern would alter. In the experiments that will be discussed below, we observe a superposition of the signals of a pure matrix and the two nucleotide contributions, indicative of a coexistence of a matrix phase and a pure nucleotide phase, likely as thin layers of nucleotide molecules.

Potential 2-dimensional nucleotide arrangements are depicted in Fig. 2. When nucleotides form a loosely packed, disordered glassy structure, as in Fig. 2b, the average distance between nucleotides is found to be 4.6 Å. The polymerization of nucleotides occurs in a condensation reaction in which phosphodiester bonds and H_2_O molecules are formed, as shown in Fig. 2c. The resulting structure resembles the backbone of an RNA molecule in which the nucleotides are found at a well defined distance of 3.4 Å, as shown in Fig. 2d. Both these distances are observed in the diffraction experiments. The volume fraction of the respective phases can be determined from the integrated intensity of the corresponding Lorentzian peak profiles, as depicted in Fig. 2a.

We will in the following discuss the experiments of nucleotides confined in lipid bilayers, thin films, NH_4_Cl salt and Montmorrillonite clay, and determine the total amount of nucleotides in the disordered, 4.6 Å, and the ordered, 3.4 Å phase.

### Nucleotides and lipid complexes

5′-adenosine monophosphate (AMP) and 5′-uridine-monophosphate (UMP) molecules were captured in a multilamellar phospholipid matrix composed of dimyristoylphosphatidylcholine (DMPC). Four systems were prepared for this part of the study: AMP/DMPC complex, UMP/DMPC and AMP/UMP/DMPC. Pure DMPC bilayers were prepared for comparison. Nucleotides and lipids were mixed in a 1:1 molar ratio.

**Figure 3:**
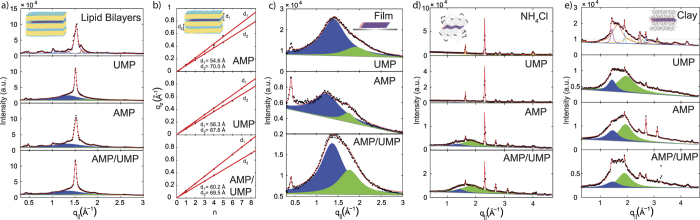
In-plane diffraction patterns of nucleotides confined in the different environments. (**a**) a pure membrane stack, DMPC with AMP, DMPC with UMP and DMPC with AMP/UMP. (**b**) Lamellar *d*-spacings for the membrane stacks in part (**a**). Two spacings are observed in the presence of nucleotides indicative of bilayers with and without a layer of nucleotides between them. (**c**) In-plane diffraction patterns of nucleotides confined in thin, solid supported films. The patterns are well described by two broad Lorentzian distributions. (**d**) NH_4_Cl complexes: The pattern for NH_4_Cl is well indexed by the known salt structure. Two broad Lorentzian distributions occur in the presence of nucleotides. (**e**) Diffraction patterns for the Montmorilllonite clay samples. Montmorillonite is a complex structure that shows a rich diffraction pattern, which agrees well with patterns in the literature. Additional broad contributions appear in the presence of the nucleotides. The peak at ~0.4 Å^−1^ is scattering from the Kapton windows of the X-ray chamber.

The pure DMPC sample in Fig. 3a shows well developed in-plane Bragg peaks along the *q*_||_-axis. The scattering features in the in-plane data are the result of the packing of the hydrocarbon chains in the membrane core and lipid head group arrangements^28^,^29^,^30^,^31^. The diffracted intensities have a distinct rod-like shape in the 2-dimensional data in Fig. S1 in the Supplementary Material, indicative of a 2-dimensional system. The out-of-plane scattering along *q*_*z*_ shows pronounced and equally spaced Bragg intensities due to the multi-lamellar organization of the membranes.

The diffraction pattern for DMPC agrees well with patterns reported in the literature for a lipid bilayer in its well ordered gel phase^29^. As reported previously, the addition of AMP leads to additional features along both out-of-plane (*q*_*z*_) and in-plane (*q*_||_) axes, indicative of 2-dimensional crystal-like AMP structures, *i.e.*, highly entangled arrangement of the AMP nucleotides encapsulated between the stacked membranes, as detailed by Toppozini *et al.*^13^. In this work we focus on the two broad contributions that occur in the presence of nucleotides, centered at 4.6 ± 0.2 and 3.4 ± 0.1 Å. The signals are well fit by Lorentzian distributions. From their angular distribution, the corresponding structures are aligned parallel to the highly oriented bilayers.

The splitting of the out-of-plane peaks is indicative of 2-dimensional layers of AMP and UMP molecules forming between the stacked membranes. The lamellar spacing *d*, *i.e.*, the distance between 2 bilayers in the membrane stack was determined from the positions of the out-of-plane peaks and is plotted in Fig. 3b. Two *d* spacings are observed indicating the coexistence of a pure lipid phase and a phase, where layers of nucleotides are confined between the bilayers.

For AMP, the difference in *d* spacings of (*d*_2_ − *d*_1_) of 15.4 Å corresponds to ~6 layers of AMP molecules when assuming a layer thickness of 2.7 Å, as determined by Toppozini *et al.* For DMPC with UMP, the difference in lamellar spacings corresponds to ~4 layers of nucleotides and in the case of AMP/UMP, 3–4 layers are determined.

### Nucleotide films

By spreading nucleotide solution directly on silicon wafers, thin films of nucleotides were prepared to simulate the effect of 2-dimensional confinement without the presence of organic molecules, salt or clay surfaces. AMP was found to have a strong affinity to crystallize and crystallites were observed in addition to the film at the rim of the dried droplet. The crystal structure of these crystallites was found to agree with crystalline AMP in the literature. AMP crystallizes in a 3-dimensional monoclinic structure P21 with unit cell dimensions of *a* = 12.77 Å, *b* = 11.82 Å, *c* = 4.882 Å and *β* = 92.24°^24^, where the adenine takes a stacked configuration. In other words, as AMP becomes increasingly concentrated, it would ordinarily form crystals with base stacking. We will show below that AMP tends to form a more glassy state in different environments, however retains the tendency to undergo stacking.

The AMP crystallites were carefully removed from the wafer and the X-ray pattern of the remaining film is shown in Fig. 3c. The absence of sharp diffraction features is indicative that no long-range order forms between the AMP molecules. The pattern is well fit by 2 broad contributions, centered at 4.6 and 3.4 Å. The uncertainties were determined from the error in fitting the peak position to 0.2 Å, respective 0.1 Å.

The scattering signals show a strong anisotropy in the 2-dimensional data in Fig. S1 in the Supplementary Material, indicative that the nucleotides take a flat, 2-dimensional orientation, parallel to the silicon wafer within the film. With a thickness of such a nucleotide layer of 2.7 Å, as determined by Toppozini *et al.*^13^, the films consist of about 700–800 nucleotide layers.

The patterns for UMP and AMP/UMP are also shown in Fig. 3c. Diffraction is well fit by two Lorentzian peak profiles. We note that the overall scattering intensity in the UMP film in this *q*-range was significantly smaller than for AMP and AMP/UMP indicative that UMP has a smaller tendency to form structures on this length scale; most of the scattering occurred at smaller *q*-values indicating of an overall more disordered structure.

### Ammonium chloride (NH_4_Cl) complexes

Salts, such as NH_4_Cl provide confinement of nucleotides during drying to an anhydrous state, as the nucleotides form thin films on the surfaces of the salt crystals. The diffraction pattern for pure NH_4_Cl is shown in Fig. 3d. A series of pronounced Bragg reflections is observed due to the formation of salt crystals. The scattered intensity is distributed over a circle indicative of a random orientation of the crystals in the sample (a so-called powder average). The peaks are well indexed by the NH_4_Cl salt structure, published for instance by Levy and Peterson^32^, with space group *Pm*3*m* and lattice constant *a* = 3.87 Å.

When nucleotides are added, two broad correlation peaks are observed in addition to the NH_4_Cl salt peaks. The position of these correlation peaks agrees with the peaks observed in bilayer complexes and nucleotide films and correspond to the distance of nucleotide molecules in the disordered phase and forming nucleotide stacks. From the coexistence of salt and nucleotide signals one can conclude that the nucleotides form layers on the surface of ammonium chloride crystals. While in the nano-films, a larger fraction of of molecules was found in the glassy state, the peak corresponding to the ordered, 3.4 Å phase is more pronounced when in contact with NH_4_Cl.

### Nucleotides with Montmorillonite clay

Clay, such as Montmorrillonite, with its layered structure provides a different type of confinement of the mononucleotides. The presence of charged surfaces was previously found to enhance the formation of short RNA oligomers from activated mononucleotides^16^. The corresponding X-ray patterns are shown in Fig. 3e. The pattern for pure clay agrees well with diffraction reported in the literature^33^. The addition of nucleotides results in the previously observed pair of broad correlation peaks centered at distances of 4.6 ± 0.2 and 3.4 ± 0.1 Å. The coexistence of pure clay and nucleotide signals is indicative that a layer of nucloetides forms on the clay surface. By taking into account the exfoliated structure of clay with its large surface area, there are about 8.5 nucleotides per square nm of clay.

## Discussion

It is plausible that some of the organic compounds required for the origin of life were delivered to Earth during late accretion^34^. An alternative source is geochemical synthesis related to volcanism^35^. It is uncertain which of the two was dominant, but it is clear that there are non-biological sources of such compounds. For instance, the Murchison carbonaceous meteorite contains over 70 amino acids, as well as most of the nucleobases used by RNA, DNA, and fatty acids from which simple membranes can be constructed^36^,^37^. However, no evidence of polymers, such as nucleic acids and peptides, has been detected, so it is likely that these polymers were necessarily synthesized by terrestrial geochemical processes rather than being delivered as such.

The basic building blocks of nucleic acids are nucleotides, composed of nucleobases linked to a sugar and phosphate. RNA is typically a single-stranded molecule with a nucleotide backbone formed by 4 different nucleotides containing the nucleobases of adenine (A), cytosine (C), guanine (G), and uracil (U). The nucleotide monomers are linked through phosphodiester bonds to form strands hundreds to thousands of nucleotides in length. RNA synthesis in living cells is usually catalyzed by an enzyme, RNA polymerase, using DNA as a template for transcription. The question is of how the first ribonucleic acids were synthesized in the absence of enzymes and pre-existing DNA.

The primary aim of the research reported here was to determine whether mononucleotides are captured and organized within different matrices, thereby promoting polymerization. As depicted in Fig. 1, the different systems investigated provide different types of confinement: lamellar membranes and oriented films provide a 2-dimensional confinement of the nucleotide molecules. Films confine nucleotides without the presence of organic molecules, or anhydrous or charged surfaces. Salt and clay provide a large surface area and mononucleotides can be confined between salt crystallites and in between the layered structure of clay. While salts provide an anhydrous environment, the clay surface is known to act as a catalyst because of its surface charges. The systems investigated here, therefore, provide a coherent set of environments to study nucleotide organization. We note that while X-ray diffraction is a powerful tool to determine molecular arrangements and distances, it is not sensitive to the formation of chemical bonds, *i.e.*, it can not prove or disprove that the nucleotides in the ordered, 3.4 Å structure have undergone the polymerization reaction to form actual RNA-like polymers. In fact, in a single cycle the yield of longer polymers has been found to be small, probably less than one percent^21^, however, with formation of dimers, trimers and tetramers.

When drying AMP, it becomes concentrated and will begin to undergo base stacking by self-assembly, then potentially start crystallization with time. In an AMP/UMP mixture, however, crystallization is not observed, instead the molecules form a glassy state, with some of the AMP/UMP forming linear arrays of stacked bases, anywhere from a few to maybe a hundred in length. We call these nucleotide stacks pre-polymers – they are ready to polymerize, but ester bond formation is a rate limiting process, which takes time to reach completion. Once formed, the polymers are relatively stable such that they accumulate every cycle until there is a steady state when they are 50 to 100 nucleotides in length.

So, what do the promoters like salt, lipid and clay do? There are several possibilities. If the nucleotides are simply dried, they become immobilized in a solid glass and are unable to diffuse, which limits their reactivity. In lipid, however, the medium is a liquid crystal within which the are free to diffuse and react. This is likely true for salts as well, as in the last stages of drying the nucleotides are concentrated in a glassy, eutectic film on the salt crystals and can form pre-polymers, then ester bonds as water activity decreases. A similar mechanism likely occurs on clay surfaces.

**Figure 4:**
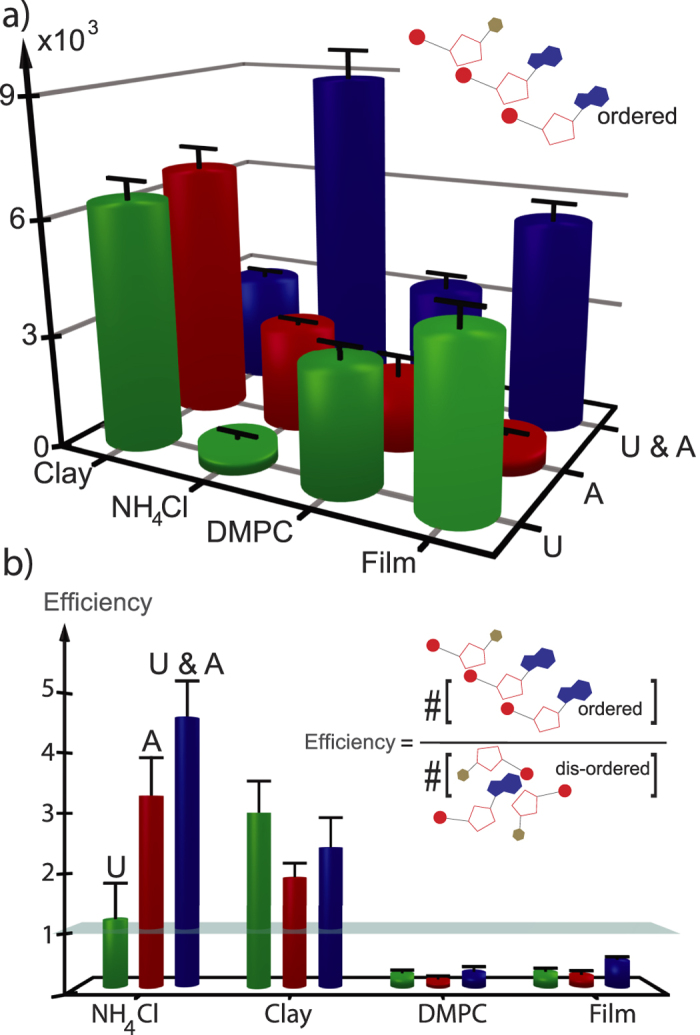
Volume fraction of ordered nucleotides in the different environments and efficiency of producing ordered stacks. (**a**) Total amount of nucleotides at a distance of 3.4 Å for clay, NH_4_Cl, DMPC and film, as determined by the integrated area of the corresponding diffraction peaks. While clay produces a large number of ordered nucleotides for pure AMP and UMP, NH_4_Cl is most productive in producing nucleotides at a distance of 3.4 Å for AMP/UMP mixtures. (**b**) The efficiency was calculated as the ratio between nucleotides at a 3.4 Å distance and nucleotides at a 4.6 Å distance. An efficiency less than 1 is indicative that more 4.6 Å nucleotides are produced while efficiencies of greater than 1 are indicative of more nucleotides at a distance of 3.4 Å. NH_4_Cl was found to be most efficient in producing nucleotides at a distance of 3.4 Å. The bars indicate the experimental error.

Our experimental results are summarized in Fig. 4. The area of the Lorentzian distribution centered at 3.4 Å is shown in Fig. 4a, representing the total amount of nucleotides found in pre-polymers at a distance corresponding to stacked base pairs in RNA polymers. Data are plotted for AMP, UMP and mixtures of AMP/UMP. Following this data, the different environments produce different amounts of ordered structures for the different types of nucleotides. While clay, for instance, is most productive for UMP, it produces a small amount of nucleotides at a distance of 3.4 Å for AMP/UMP mixtures, only. Ammonium salt on the other hand was found to be most productive for AMP/UMP mixtures while it produced small amounts of ordered structures for pure AMP or pure UMP.

While Fig. 4a plots the total amount of ordered nucleotides in the different environments, the “efficiency” in producing ordered 3.4 Å over disordered 4.6 Å structures, as determined by the ratio between the integrated area of the corresponding Lorentzian distributions, is plotted in Fig. 4b. Here, a value of greater than 1 means that this environment produces more ordered than disordered nucleotides; a value less than 1 is indicative that more disordered nucleotides are formed. Also in this representation, NH_4_Cl is most effective in producing 3.4 Å structures in AMP/UMP mixtures. While Montmorillonite clay is still effective, lipid bilayers and films produce more disordered nucleotides.

**Figure 5:**
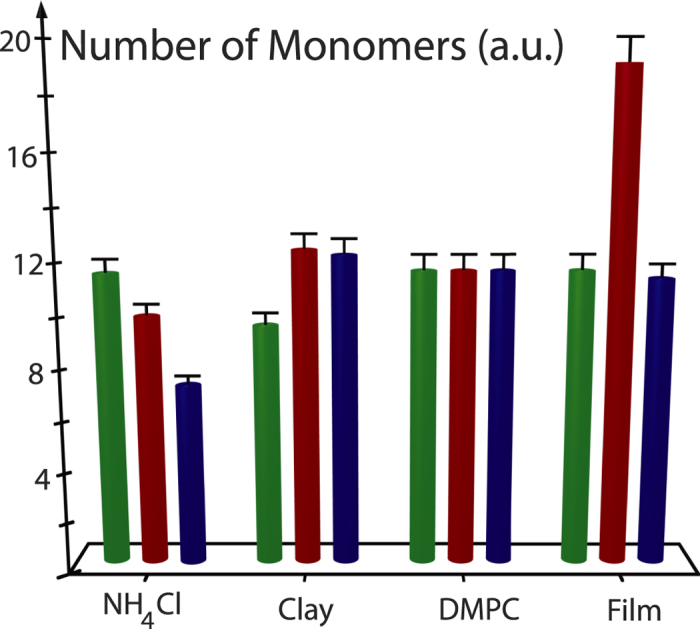
Length of the nucleotide stacks from experiments. Length of the pre-polymer nucleotide stacks as determined from the peak width of the corresponding diffraction peaks using Scherrer’s equation. The bars indicate the experimental error.

The average length, *L*, of the nucleotide stacks can be estimated from the widths of the corresponding correlation peaks by applying Scherrer’s equation^38^:


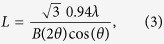


where *λ* is the wavelength of the X-ray beam, *θ* is the diffraction angle and *B*(2*θ*) is the width of the correlation peak in radians. Eq. (3) was corrected to calculate the correct length for a 1-dimensional structure by multiplying by 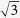. The corresponding values are plotted in Fig. 5. Based on these values the average length of a nucleotide stack was found to be about 10 monomers.

An important question is the structure corresponding to the 3.4 Å spacing. There are several possibilities. On the one hand the nucleotides can form pure AMP and pure UMP strands. Alternatively, they can form randomly organized, mixed stacks (*AAUAUUUAUAAUAUAUAUU*). For the first case, we would assume that the pre-polymer concentration in an AMP/UMP mixture is the sum of the pre-polymer configuration for pure AMP and pure UMP, *i.e.* the sum of the *A* and *U* columns in Fig. 4a. However, while clay produces about the same amounts of nucleotides at a distance of 3.4 Å for AMP and UMP, it produces significantly fewer in AMP/UMP mixtures; NH_4_Cl produces a significantly higher amount of pre-polymers for AMP/UMP mixtures than for the AMP and UMP.

The rate constants *c*_3_, *c*_4_, *c*_5_ and *c*_6_ in Eqs. (1) can be determined from the the data in Fig. 4a. The constants for the formation of *AA* and *UU* pairs can directly be determined by the results from pure AMP and pure UMP systems. In the mixed AMP/UMP systems, however, *AA*, *UU* and *AU* pairs can form such that additional arguments are necessary.

All possible configurations for a stack of, for instance, three elements are:


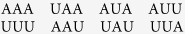


The probability that a stack of three elements exists in one of the separate states (only *A* or only *U*) is 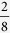. The probability for a mixed phase on the other hand is calculated to 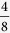. For a longer stack with *n* elements, these probabilities are given by 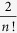 and 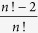. This leads to 
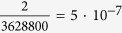
 and 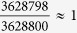 for the measured length of 10 elements in Fig. 5. As the probability for the formation of *AA* and *UU* pairs in mixed AMP/UMP systems is, therefore, negligibly small, the parameters *c*_3_, *c*_4_, *c*_5_ and *c*_6_ in Eqs. (1) can, in good approximation, be determined from the measurements by the following relations:


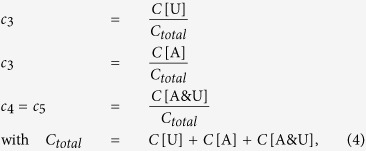


where *C*[⋅] is the total amount of the corresponding structure in Fig. 4a. The calculated values for *c*_3−6_ are given in Table 1.

**Figure 6:**
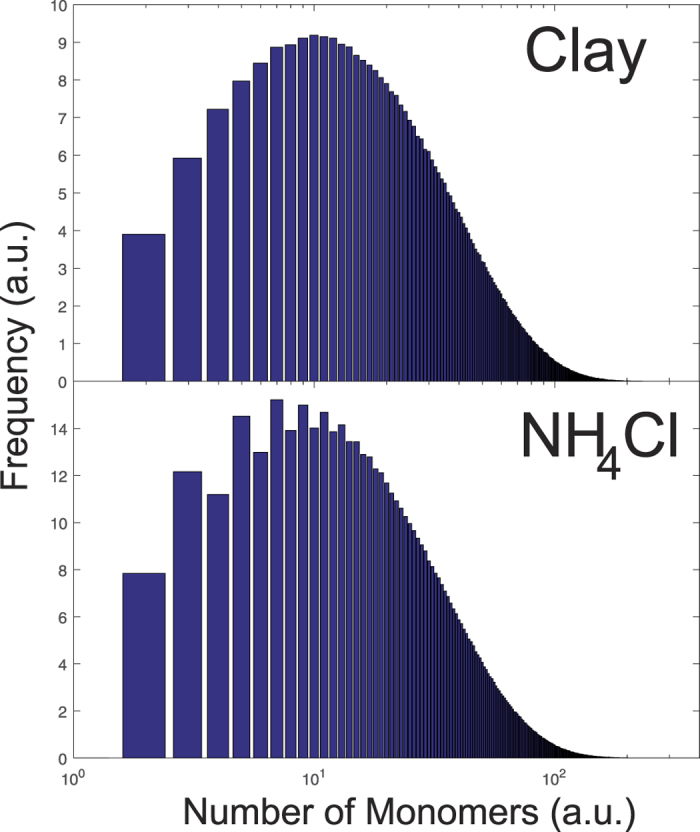
Length distribution from simulations. Length distribution for different simulations using the experimentally determined parameters for clay and NH_4_Cl. The average nucleotide length is determined from a Lorentzian distribution to ~12 (clay) and ~7 (NH_4_Cl) nucleotides, in good agreement with the experiments.

The parameters in Table 1 were then used to run the Monte-Carlo simulations. Data of 1000 iterations were collected to calculate the length distribution of the resulting polymers. The results for clay and NH_4_Cl are shown in Fig. 6. The centers of the distribution are determined at ~7 for NH_4_Cl and ~12 for clay, in good agreement with the experimental findings. The simulations also indicate the presence of a small number of longer stacks up to about 100 monomers.

**Figure 7:**
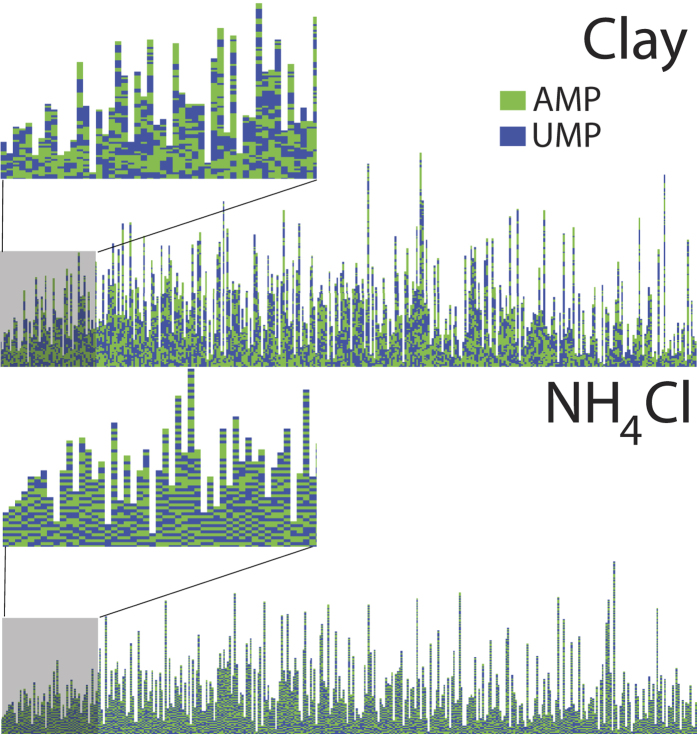
Pre-polymer configurations for two different materials. Shown are simulation snapshots and sample structures of the pre-polymers for clay and NH_4_Cl obtained from the simulations. Large *AA* and *UU*, only, segments are observed in clay while NH_4_Cl strongly favors the formation of *AU* pairs and, therefore, was found to form more uniformly mixed stacks.

Snapshots of the some of the nucleotide stacks produced in a single iteration of the simulations are depicted in Fig. 7 for clay and NH_4_Cl. While the formation of pairs was found to be less favorable in clay, more *AU* pairs were found to form in the presence of NH_4_Cl. Accordingly, the nucleotide stacks in Fig. 7a show large domains of *AA* and *UU* only pairs, while the ammonium salt leads to a well mixed *A* and *U*, alternating sequence in part b).

While Mungi and Rajamani^21^ reported the formation of RNA-like oligomers through lipid-assisted non-enzymatic synthesis they observed that the resultant polymers can lose their bases through the reaction process resulting in abasic sites. Unlike RNA, these “prebiotic phosphodiester polymers” lack information-carrying capability. The formation of abasic sites in the pre-polymers observed in this work has to be tested in future experiments.

A comment is in order. Toppozini *et al.*^13^ recently reported that AMP molecules form a highly ordered nucleotide phase when confined between phospholipid bilayers. The authors observed a series of well defined Bragg peaks in the in-plane diffraction patterns. While we could reproduce the previous experiments in DMPC/AMP complexes we did not find evidence for this highly entangled phase in UMP or AMP/UMP mixtures. It can be speculated that the high affinity of AMP to crystallize drives the formation of the 2-dimensional crystallites in the presence of membranes. We did also not find evidence for this AMP structure in films, salt or clay such that this phase seems particular to lipid membrane complexes.

In this paper we present evidence that the more commonly formed nucleotide structures are the disordered glassy phase and the ordered 3.4 Å phase, characterized by two broad scattering contributions. These phases are also observed in DMPC/AMP complexes and coexist with the highly entangled phase reported by Toppozini *et al.* There are, therefore, two pathways for AMP molecules to potentially form RNA-like polymers in contact with phospholipid bilayers.

Future experiments will address mixtures of all 4 nucleotides and in particular combinations of the environments studied in this work to mimic the conditions found at hydrothermal sites. As the polymerization is driven by a condensation reaction, high temperatures and low hydrations should promote polymerization and cycling through temperature and humidity should lead to the formation of longer and longer polymer chains in the experiment.

## Conclusion

We studied the organization of adenosine and uridine monophosphate in different environments. AMP and UMP and AMP/UMP mixtures were prepared with phospholipid bilayers, as thin films, with ammonium chloride salt and Montmorillonite clay. The different systems provide different types of confinement: lamellar membranes and oriented films provide a 2-dimensional confinement of the nucleotide molecules. Films confine nucleotides without the presence of organic molecules, or anhydrous or charged surfaces. Salt and clay provide a large surface area and mononucleotides can be confined between salt crystallites and in between the layered structure of clay. While salts provide an anhydrous environment, the clay surface is known to act as a catalyst because of its surface charges. These systems provide a coherent set of environments to study nucleotide organization.

The molecular organization of the nucleotides was studied using X-ray diffraction. Two contributions of the nucleotides were observed in the X-ray patterns: nucleotides at a distance of 4.6 Å were found to form a disordered, glassy phase. The signal at 3.4 Å was assigned to the formation of pre-polymers, *i.e.*, to stacks of nucleotides at the distance of base pairs in RNA. The fraction of nucleotides in the two states in the different environments was determined from the integrated peak intensities of the corresponding scattering signals. An average stack length of these pre-polymers of 10 nucleotides was found in the experiments.

The different environments were found to produce different amounts of nucleotides at a distance of 3.4 Å for AMP, UMP and AMP/UMP mixtures. While NH_4_Cl and clay were found to be most efficient in producing nucleotides at the base pair distance, nucleotides confined by lipid bilayers and in thin films were mostly found in the disordered state. Rate constants for the formation of *AA*, *UU* and *AU* pairs were determined from the experiments and used in Monte-Carlo simulations. Structure of the polymer stacks and stack lengths were determined from these simulations. We argue that the monomers in these pre-polymers can be linked into long strands when ester bonds are synthesized by cyclic fluctuations in temperature and hydration.

## Additional Information

**How to cite this article**: Himbert, S. *et al.* Organization of Nucleotides in Different Environments and the Formation of Pre-Polymers. *Sci. Rep.* **6**, 31285; doi: 10.1038/srep31285 (2016).

## Supplementary Material

Supplementary Information

## Figures and Tables

**Table 1:** Rate constants, as determined from the experiments.

	*c*_3_	*c*_4_	*c*_5_	*c*_6_
Clay	0.350	0.367	0.141	0.141
NH_4_Cl	0.012	0.129	0.429	0.429
DMPC	0.273	0.149	0.289	0.289
Film	0.271	0.425	0.343	0.343

Rate constants *c*_3_, *c*_4_, *c*_5_ and *c*_6_, as determined from the integrated peak intensities in Fig. 4. The constants were calculated using Eq. (4).
