# Supplementary material for: Organization of Nucleotides in Different Environments and the Formation of Pre-Polymers
Source: Sci Rep. 2016 Aug 22;6:31285. doi: 10.1038/srep31285 (PMC4992878; doi:10.1038/srep31285)
Supplement: Supplementary Information [file srep31285-s1.pdf]

Supplementary Material to: Organization of Nucleotides in Different Environments and the Formation of Pre-Polymers

Sebastian Himbert,[1,2,3] Mindy Chapman,[1,2] David W. Deamer,[4] and Maikel C. Rheinstädter[1,2,*]

[1]*Department of Physics and Astronomy, McMaster University, Hamilton, Ontario, Canada*

[2]*Origins Institute, McMaster University, Hamilton, L8S 4M1, Canada*

[3]*Saarland University, Department of Experimental Physics, 66123 Saarbrücken, Germany*

[4]*University of California, Department of Biomolecular Engineering, Santa Cruz, 95064, USA*

[*] rheinstadter@mcmaster.ca; Department of Physics and Astronomy, McMaster University, ABB-241, 1280 Main Street West, Hamilton, Ontario L8S 4M1, Canada; Phone: +1-(905)-525-9140-23134, Fax: +1-(905)-546-1252

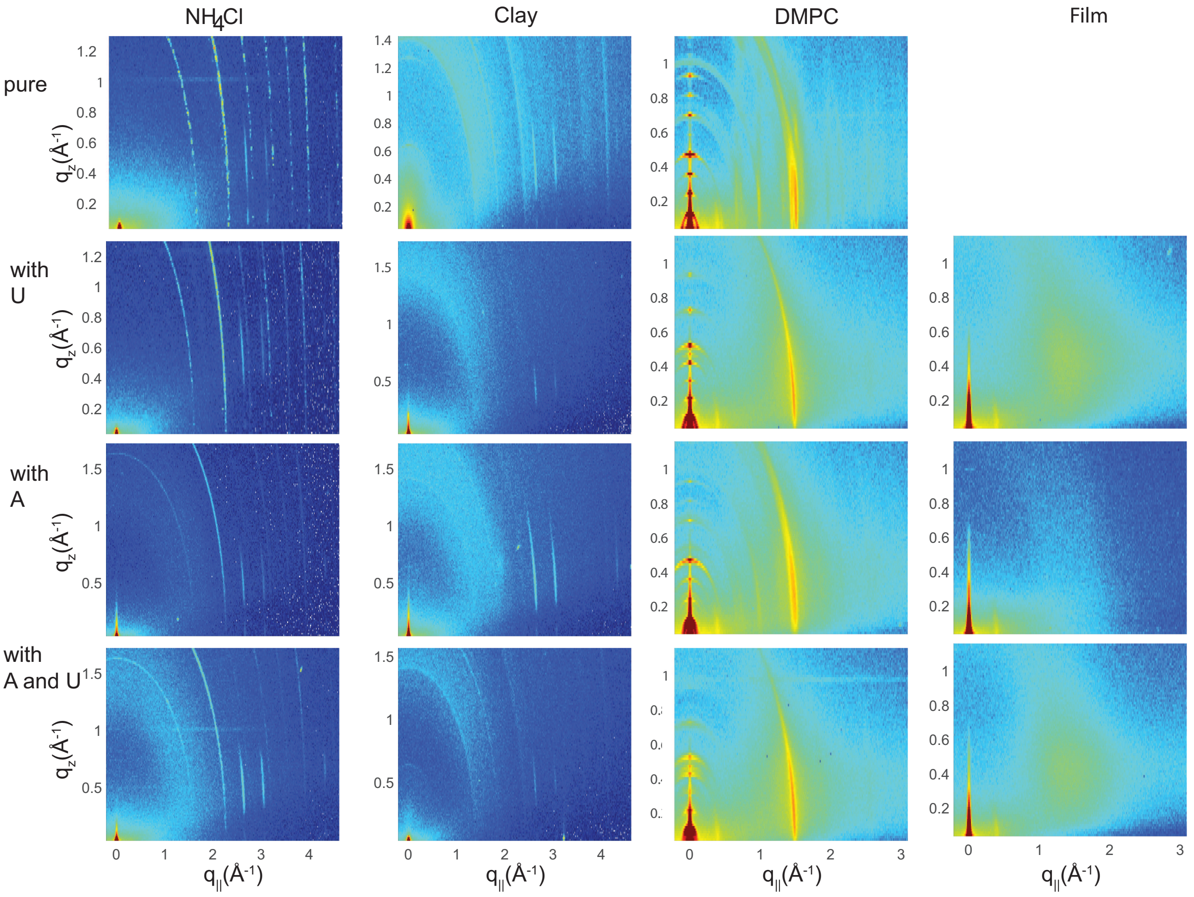

FIG. S1. **Two-dimensional X-ray diffraction Data for all Samples.** Two-dimensional X-ray diffraction for AMP, UMP and AMP/UMP mixtures with: NH_4Cl salt, Montmorillonite clay, lipid bilayers made of DMPC, and thin films.
